# Supplementary material for: Deletion of wfs1 Impairs Oligodendrocyte Precursor Cells Dorsal Distribution and Myelination Through the wfs1-hmgcs1 Axis in Zebrafish
Source: Biology (Basel). 2026 Aug 16;15(16):1402. doi: 10.3390/biology15161402 (PMC13510151; doi:10.3390/biology15161402)
Supplement: Supplementary file 1 [file biology-15-01402-s001.zip › File S1-Transcriptome sequencing data and differentially expressed genes (DEGs) in wfs1-- zebrafish at 5 dpf/1.GO/wfs1KOvsWT/GSEA/WT/REGULATION_OF_DNA_REPAIR/REGULATION_OF_DNA_REPAIR(GO_0006282).html]

Details for gene set REGULATION\_OF\_DNA\_REPAIR(GO:0006282)[GSEA]


|  || Dataset | deg.sample |
| Phenotype | sample.cls |
| Upregulated in class | WT |
| GeneSet | REGULATION\_OF\_DNA\_REPAIR(GO:0006282) |
| Enrichment Score (ES) | -0.39498058 |
| Normalized Enrichment Score (NES) | -1.77113 |
| Nominal p-value | 0.0 |
| FDR q-value | 0.30897525 |
| FWER p-Value | 0.717 |
Table: GSEA Results Summary

  

Fig 1: Enrichment plot: REGULATION\_OF\_DNA\_REPAIR(GO:0006282)
  
Profile of the Running ES Score & Positions of GeneSet Members on the Rank Ordered List

  

| PROBE | DESCRIPTION   (from dataset) | GENE SYMBOL | GENE\_TITLE | RANK IN GENE LIST | RANK METRIC SCORE | RUNNING ES | CORE ENRICHMENT || 1 | 641417 | zgc:123335 |  |  | 1006 | 2.087 | 0.0440 | No |
| 2 | 100333845 | brcc3 |  |  | 2429 | 1.197 | 0.0427 | No |
| 3 | 406820 | actr2a |  |  | 3314 | 0.969 | 0.0501 | No |
| 4 | 571385 | eya3 |  |  | 4734 | 0.713 | 0.0314 | No |
| 5 | 393160 | tigarb |  |  | 6007 | 0.585 | 0.0126 | No |
| 6 | 108179053 | fancb |  |  | 6106 | 0.576 | 0.0304 | No |
| 7 | 449820 | abraxas2a |  |  | 6237 | 0.564 | 0.0467 | No |
| 8 | 100003611 | si:ch211-26b3.2 |  |  | 6760 | 0.514 | 0.0490 | No |
| 9 | 503732 | rtel1 |  |  | 8771 | 0.383 | -0.0003 | No |
| 10 | 572849 | suv420h1 |  |  | 9019 | 0.366 | 0.0052 | No |
| 11 | 322007 | usp1 |  |  | 9207 | 0.353 | 0.0121 | No |
| 12 | 550474 | babam2 |  |  | 9509 | 0.334 | 0.0148 | No |
| 13 | 100149820 | si:ch211-134m17.9 |  |  | 10313 | 0.284 | -0.0002 | No |
| 14 | 30436 | eya1 |  |  | 10542 | 0.270 | 0.0025 | No |
| 15 | 797792 | pargl |  |  | 10772 | 0.257 | 0.0046 | No |
| 16 | 797342 | helb |  |  | 11401 | 0.222 | -0.0071 | No |
| 17 | 450064 | zgc:101664 |  |  | 11589 | 0.212 | -0.0053 | No |
| 18 | 334682 | wdr48b |  |  | 11831 | 0.199 | -0.0057 | No |
| 19 | 692304 | dek |  |  | 13170 | 0.132 | -0.0429 | No |
| 20 | 678635 | parpbp |  |  | 14782 | 0.056 | -0.0915 | No |
| 21 | 447819 | eya2 |  |  | 14981 | 0.047 | -0.0960 | No |
| 22 | 664696 | tigara |  |  | 15440 | 0.025 | -0.1094 | No |
| 23 | 554861 | actr2b |  |  | 15691 | 0.012 | -0.1168 | No |
| 24 | 557043 | mb21d1 |  |  | 17109 | -0.037 | -0.1600 | No |
| 25 | 407707 | terf2ip |  |  | 18052 | -0.078 | -0.1867 | No |
| 26 | 563643 | mms19 |  |  | 18383 | -0.093 | -0.1937 | No |
| 27 | 799320 | znf365 |  |  | 18613 | -0.104 | -0.1972 | No |
| 28 | 101882450 | slf1 |  |  | 18920 | -0.119 | -0.2025 | No |
| 29 | 405856 | wrnip1 |  |  | 19917 | -0.162 | -0.2279 | No |
| 30 | 334627 | ubr5 |  |  | 21557 | -0.244 | -0.2705 | No |
| 31 | 404050 | rnf8 |  |  | 22378 | -0.288 | -0.2858 | No |
| 32 | 393313 | ube2nb |  |  | 22491 | -0.295 | -0.2786 | No |
| 33 | 492470 | spire2 |  |  | 22636 | -0.304 | -0.2721 | No |
| 34 | 751761 | fam168a |  |  | 22893 | -0.319 | -0.2686 | No |
| 35 | 795748 | rnf169 |  |  | 23757 | -0.377 | -0.2821 | No |
| 36 | 334081 | rnf168 |  |  | 23936 | -0.388 | -0.2736 | No |
| 37 | 436684 | otub1a |  |  | 24435 | -0.416 | -0.2742 | No |
| 38 | 563218 | rmi2 |  |  | 24602 | -0.422 | -0.2641 | No |
| 39 | 445296 | babam1 |  |  | 25381 | -0.473 | -0.2714 | No |
| 40 | 436773 | otub1b |  |  | 29318 | -1.006 | -0.3585 | Yes |
| 41 | 564866 | trip12 |  |  | 29348 | -1.013 | -0.3227 | Yes |
| 42 | 30590 | tp53 |  |  | 30325 | -1.401 | -0.3026 | Yes |
| 43 | 557962 | spire1a |  |  | 30915 | -1.880 | -0.2530 | Yes |
| 44 | 541552 | eya4 |  |  | 31710 | -3.769 | -0.1414 | Yes |
| 45 | 100001877 | si:ch211-127m7.2 |  |  | 31743 | -4.059 | 0.0046 | Yes |
Table: GSEA details
[plain text format]

  

Fig 2: REGULATION\_OF\_DNA\_REPAIR(GO:0006282)
  
Blue-Pink O' Gram in the Space of the Analyzed GeneSet

  

Fig 3: REGULATION\_OF\_DNA\_REPAIR(GO:0006282): Random ES distribution
  
Gene set null distribution of ES for
 **REGULATION\_OF\_DNA\_REPAIR(GO:0006282)**
